# Building Surgical Capacity in Resource‐Limited Settings Through Innovative Laparoscopy Training: A Systematic Review of Educational Interventions

**DOI:** 10.1002/wjs.70423

**Published:** 2026-05-15

**Authors:** Ali Yasen Mohamedahmed, Shafquat Zaman, Safeya Mohammed, Seddig Adam Jebril Saleh, Mohammed A. Adam, Johnelize Louw, Tiffany E. Chao, Kathryn Chu

**Affiliations:** ^1^ Centre for Global Surgery Stellenbosch University Stellenbosch South Africa; ^2^ Department of Hepatobiliary and pancreatic surgery University Hospitals of Leicester Leicester UK; ^3^ Department of Colorectal and General Surgery Worcestershire Acute Hospitals NHS Trust Worcestershire Royal Hospital Worcester UK; ^4^ College of Medical and Dental Sciences School of Medicine University of Birmingham Birmingham UK; ^5^ Department of General Surgery Ibrahim Malik Teaching Hospital Khartoum Sudan; ^6^ Department of General Surgery University of Al Fashir Al Fashir Sudan; ^7^ Global Surgery Lab Sudanese Consortium of Surgical Development Khartoum Sudan; ^8^ School of Public Heath Imperial College London London UK; ^9^ Department of Surgery Stanford University School of Medicine Stanford California USA

**Keywords:** healthcare equity, laparoscopic surgery, low‐ and middle‐income countries, training

## Abstract

**Background:**

Laparoscopic surgery offers substantial benefits compared to open surgery, yet access to structured laparoscopic training remains limited in low‐ and middle‐income countries (LMICs). This systematic review aimed to synthesize evidence on training interventions, methods, and outcomes of laparoscopic education programs in LMICs.

**Methods:**

A systematic search of PubMed, Embase, Scopus, and Web of Science was performed from 1990 to September 2025, including studies that reported laparoscopic training programs conducted in LMICs as defined by the World Bank classification. Data were extracted on study setting, training method, participant characteristics, and outcomes. Due to heterogeneity of study designs and interventions, a narrative synthesis was conducted.

**Results:**

Nineteen studies were included, spanning Africa, Latin America, Asia, and the Caribbean. Training methods were diverse, encompassing low‐cost simulators, telesimulation and telementoring, structured curricula, international collaborations, and virtual or remote learning. Participant groups ranged from small cohorts of residents and rural surgeons to large‐scale programs training thousands of practitioners. Reported outcomes consistently demonstrated improved technical skills, increased knowledge and confidence, high participant satisfaction, and evidence of feasibility and cost‐effectiveness. Longitudinal implementation studies in Ghana and Ethiopia highlighted progressive adoption of laparoscopy with increasing independence of local surgeons. Barriers identified included limited access to equipment, lack of simulation laboratories, and insufficient integration into formal curricula.

**Conclusions:**

Laparoscopic training in LMICs is feasible, effective, and increasingly diverse in delivery models. Innovative low‐cost simulators, telesimulation, and structured curricula have proven particularly successful in overcoming resource constraints. Expansion of sustainable context‐appropriate training programs is essential to strengthen surgical capacity in LMICs.

## Graphical abstract

This work has been conducted as partial fulfillment of the Global Surgery master's degree, Stellenbosch University, South Africa. Moreover, it has not been previously published, and the manuscript (including related data) is not currently under consideration elsewhere.

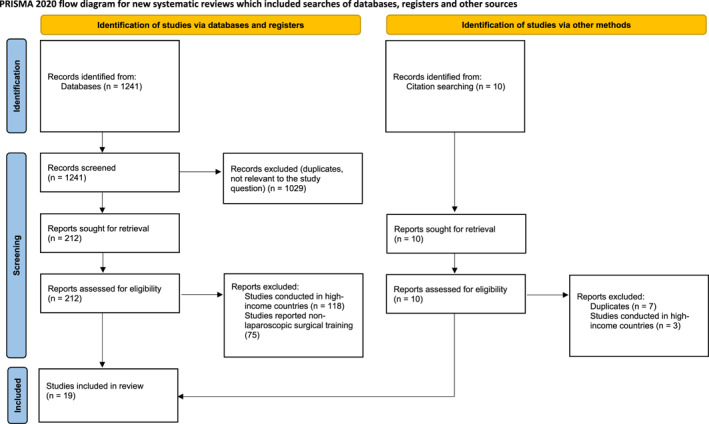

## 1 Introduction

The global disparity in healthcare remains a pressing and widening problem, driven by socioeconomic status, the emergence of new challenges (such as the COVID‐19 pandemic), environmental conditions, and technological factors [1, 2]. These inequalities are particularly evident in surgical practice, where disparities in access to safe, timely, and affordable surgical care contribute significantly to poorer outcomes and higher mortality in low‐resource settings [3].

Surgical practice has advanced significantly with the introduction of minimally invasive techniques, which offer well‐established benefits over open surgery for both patients and healthcare systems [4]. However, access to these technologies remains largely limited to high‐income settings. In low‐ and middle‐income countries (LMICs), shortages of trained personnel, inadequate infrastructure, and high equipment costs restrict adoption, contributing to ongoing disparities in surgical outcomes and postoperative morbidity [3, 5]. Common entry‐level laparoscopic procedures that are highly transferable to LMIC settings include laparoscopic cholecystectomy and appendectomy [6, 7, 8]. High‐quality evidence, including systematic reviews and Cochrane analyses, demonstrates that these procedures are associated with reduced postoperative pain, shorter hospital stay, faster return to normal activity, and lower wound complication rates compared to open surgery [9, 10]. In addition, laparoscopic approaches may offer health economic benefits through reduced length of stay and earlier return to work, particularly in resource‐constrained settings [6].

Establishing laparoscopic surgery in low‐ and middle‐income countries (LMICs) is likely to yield significant positive healthcare benefits. Barriers to the uptake and adoption of laparoscopic surgery in these economies include costs of equipment and maintenance, resource availability, unreliable infrastructure, resistance to change, and a lack of standardized and effective training pathways [11, 12, 13]. There is often a degree of reliance on foreign instructors, which creates logistical challenges. Language barriers, nonstandardized curricula, and a lack of validated assessment tools provide further challenges to skill acquisition and development in developing countries [11].

Laparoscopic training in LMICs has received considerable attention, with several international groups seeking to address these inequalities through training courses [14]. For instance, the Society of American Gastrointestinal and Endoscopic Surgeons (SAGES) introduced the low‐cost simulation‐based international, collaborative Global Laparoscopic Advancement Program (GLAP). This seeks to standardize laparoscopic education and encourage the implementation of validated Fundamentals of Laparoscopic Skills (FLS) testing in host nations [15]. Additionally, through its structured education program, it aims to reduce reliance on foreign educators [11]. However, LMICs are highly heterogeneous [6]. Although laparoscopic and even robotic surgery are well established in upper‐middle‐income countries and private or urban centers, access remains extremely limited in rural and district‐level hospitals in many low‐income settings, where even basic surgical services may be unavailable. This disparity highlights the need for context‐specific approaches to laparoscopic training and implementation [3, 5, 6]. Improving laparoscopic training in LMICs will enhance overall access to quality surgical care and improve patient outcomes, especially in settings with poor sanitation and limited resources [6, 16, 17].

We conducted a systematic review of the literature to assess the feasibility and effectiveness of various laparoscopic training methods to enhance access to and familiarity with laparoscopic surgery in LMICs.

## 2 Methods

This systematic review was conducted in accordance with the Preferred Reporting Items for Systematic Reviews and Meta‐Analyses (PRISMA) 2020 guidelines [18, 19]. The review protocol was developed a priori to ensure systematic and transparent reporting of all methodological processes and registered in the PROSPERO database (identification number CRD42024593992).

### 2.1 Search Strategy

A comprehensive and systematic search was conducted to identify studies reporting laparoscopic training in low‐ and middle‐income countries (LMICs) as defined by the World Bank classification at the time of each study. Searches were performed in major bibliographic databases, including PubMed, Embase, Scopus, and Web of Science, covering the period from inception to September 2025. The search strategy combined terms related to laparoscopy, training, simulation, education, and low‐ and middle‐income countries. References of included articles and relevant reviews were also screened to identify additional studies.

### 2.2 Eligibility Criteria

Studies were considered eligible for inclusion if they satisfied all of the following:

- Reported on laparoscopic training interventions or educational programs
- Conducted in LMICs (according to the World Bank classification)
- Reported either quantitative or qualitative outcomes related to skills acquisition, trainee performance, satisfaction, or program feasibility
- Included surgical trainees, practizing surgeons, or multidisciplinary healthcare staff involved in laparoscopic training/procedures

All study designs were eligible, including pilot studies, feasibility studies, prospective cohorts, cross‐sectional surveys, mixed‐methods evaluations, and descriptive reports. Reviews and commentaries were included only if they contributed data on training practices or barriers. Studies conducted exclusively in high‐income countries were excluded.

### 2.3 Study Selection

All records retrieved from the database search were imported and duplicates were removed. Titles and abstracts were screened independently by two reviewers to identify potentially relevant studies. The full texts of the selected articles were then reviewed against our eligibility criteria. Disagreements were resolved through discussion until a consensus was reached.

### 2.4 Data Extraction

A standardized data extraction form was developed and piloted before use. Information extracted included: author and year of study, country of origin, study design, training method, participant characteristics, and reported outcomes. Where available, objective outcome measures, such as Fundamentals of Laparoscopic Surgery (FLS) scores, Objective Structured Assessment of Technical Skills (OSATS), or cumulative sum (CUSUM) analysis, were recorded. Training interventions were further categorized as simulation‐based, hybrid (simulation and mentorship), or clinical (patient‐based training involving supervised procedures). Data extraction was performed independently by two reviewers, with discrepancies resolved through discussion with the authorship team.

### 2.5 Data Synthesis

Given the heterogeneity of study designs, interventions, and reported outcomes, a narrative synthesis was performed and meta‐analysis was precluded. Studies were grouped by geographic region, training method, and type of participant. Outcomes were summarized thematically as improvements in knowledge, skills, performance, participant satisfaction, or program feasibility. Quantitative results were presented descriptively, without formal meta‐analysis.

## 3 Results

### 3.1 Countries and Study Settings

**FIGURE 1:**
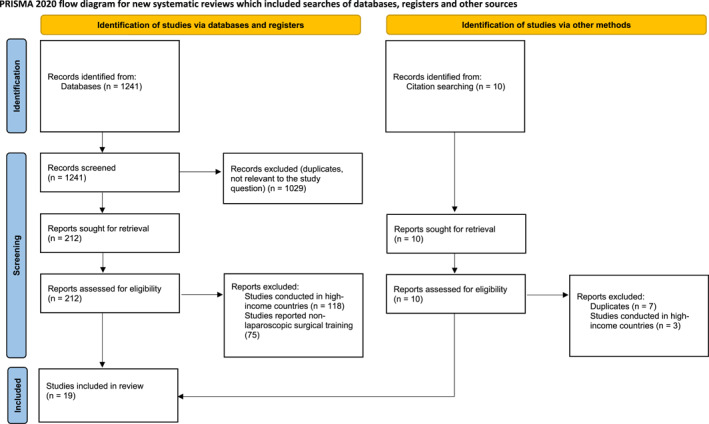
PRISMA flow diagram.

**FIGURE 2:**
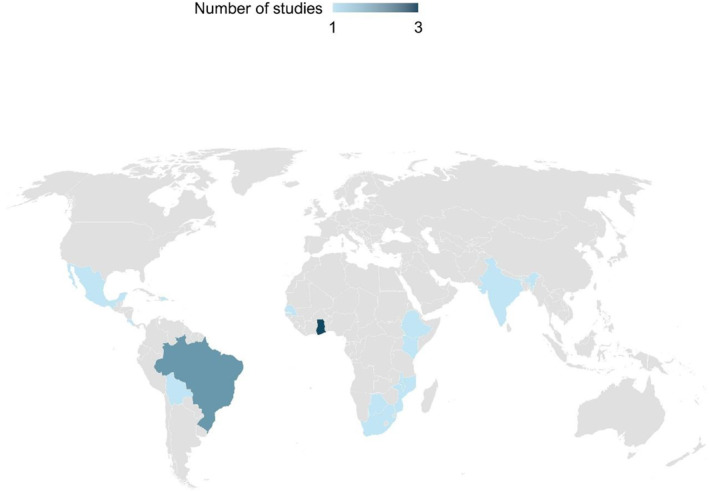
World map showing the distribution of included studies.

The systematic literature search yielded 1251 articles (Figure 1). Of these, nineteen studies [13, 20, 21, 22, 23, 24, 25, 26, 27, 28, 29, 30, 31, 32, 33, 34, 35, 36, 37] were deemed eligible for inclusion in this review, representing a diverse range of LMICs across Africa, Asia, Latin America, and the Caribbean (Figure 2). Studies from the African continent were conducted in Ghana [21, 27, 28], Kenya [29], South Africa [30], Ethiopia [31], Senegal [33], Botswana [35], Mozambique [13], and Malawi [36]. Latin American contributions included studies from Costa Rica and Mexico [23], Brazil [24, 32], Mexico [25], and Bolivia [37]. Additional studies originated from India [22] and the Dominican Republic [26]. Study designs were heterogeneous and ranged from small pilot and feasibility studies [21, 22, 23] to prospective cohort analyses [21, 33], cross‐sectional surveys [34], mixed‐methods evaluations [37], and descriptive program reports [13, 24, 30]. Characteristics of the included studies are shown in Table 1.

**TABLE 1:** Characteristics of the included studies, settings, training methods, and outcomes.

Author and year	Country	Type of study	Training method and setting	Participants	Outcome
Alfa‐Wali and Osaghae (2017) [16]	Multiple LMICs	Narrative review	Review of practice and innovative low‐cost methods Setting: Nonspecific	Not specified	Describes barriers, safety concerns, and educational gaps; notes innovative training methods but calls for more data
Andreatta et al. (2014) [17]	Ghana	Pilot study	Low‐cost locally sourced simulation‐based program using box trainers Setting: Simulation‐based training	18 faculty and house officers	Improved tissue‐handling skills; high feasibility and cost‐effectiveness (< 30 USD/participant)
Aruparayil et al. (2021) [18]	India	Observational feasibility study	Structured 3‐day simulation‐based gasless laparoscopy training Setting: Simulation‐based training	7 rural surgeons	Improved knowledge and FLS scores; OSATS and GOALS scores indicated competency; and program cost‐effective
Asfaw et al. (2023) [19]	Costa Rica, Mexico	Feasibility study	Virtual telesimulation via global laparoscopic advancement program (GLAP) Setting: Telesimulation	36 (16 attendings and 20 residents)	Significant FLS performance improvement; 100% satisfaction; scalable; and low‐cost
Crema et al. (2022) [20]	Brazil	Descriptive report	In‐person training at IRCAD Barretos; includes simulation and real surgical scenarios Setting: Simulation and real patient‐based training	Approximately 12,000 trained over 10 years	High participant satisfaction; emphasizes the importance of simulation and curriculum integration
Escamirosa et al. (2015) [21]	Mexico	Validation study	Homemade iPhone‐based laparoscopic trainer Setting: Telesimulation	20 surgical residents	No significant performance difference from standard trainer; affordable and portable option
Fisher et al. (2022) [22]	Dominican Republic	Quasi‐Experimental	Simulation center with weekly structured sessions and 3 FLS tasks Setting: Simulation and real patient‐based training	11 surgical residents	Significant improvement in precision cutting and knot tying; successful program integration
Gyedu et al. (2014) [23]	Ghana	Retrospective review/program implementation report	On‐site surgeon champion, outside expert mentoring (yearly visits), FLS model exposure, OR team training, and equipment donation/repair Setting: Simulation and real patient‐based training	8 general surgeons and theater staff at Komfo Anokye teaching hospital	25 laparoscopic cholecystectomies (2010–2012); outcomes measures were operation time, length of hospital stay, and postoperative complications. Patient satisfaction was high. Demonstrated feasibility, leadership, and surgeon champion key.
Kang et al. (2020) [24]	Ghana	Prospective implementation study (32‐month report)	Long‐term onsite training program. Two‐week simulation workshops with box trainers, animal labs, ongoing on‐the‐job mentoring, permanent skills lab, and overseas fellowships. Setting: Simulation and real patient‐based training	97 doctors (simulation training), 32 animal lab trainees, and 11 nurses trained.	82 laparoscopic procedures (2017–2019, including cholecystectomy, appendectomy, gynecologic, pediatric, and urology). The proportion of primary operators increased from 0% to 79.4% of local surgeons. > 95% trainee satisfaction. No conversions, no technical complications or mortalities. High technical independence achieved, sustainable training program established.
Long et al. (2014) [25]	Kenya	Pilot implementation study	Low‐cost laparoscopic curriculum ($50 materials). Adapted University of Kentucky validated curriculum. Trainer boxes and personal laptops, supervised, then independent practice. Setting: Simulation and real patient‐based training	10 general surgery residents (Tenwek hospital, Bomet)	Initially unable to complete timed tasks. After 3 weeks of independent practice, residents successfully performed cannulation drill (avg 80 vs. 110s baseline), peg transfer, rope pass (all improved, reduced errors). Feasible, very low‐cost, provided critical exposure. Highlighted need for sustained curriculum for retention
Mangray et al. (2023) [26]	South Africa	Descriptive study	Developed Grey's laparoscopic suturing course (GLSC), an innovative low‐cost simulation model during COVID‐19, focused on intracorporeal suturing using a simulation lab, and a structured curriculum. Setting: Simulation and real patient‐based training	47	Significant improvement in posttest scores (from 50% to 88%); all participants achieved competent suturing skills, resulting in a unanimous recommendation.
Morrow et al. (2016) [27]	Ethiopia	Prospective cohort study	Adapted the laparoscopic skills curriculum with simulation‐based tasks. Training was conducted in 6 courses with task completion and survey feedback. Setting: Simulation‐based training	88	Improved task completion time, positive feedback, and curriculum adopted by local faculty.
Nácul et al. (2015) [28]	Brazil	Review article	Critical review of residency training using psychomotor simulation models such as fundamentals of laparoscopic surgery (FLS) and laparoscopic surgical skills. Setting: Simulation and real patient‐based training	Not specified	Highlighted insufficiency in residency lap training; recommended simulation‐based structured learning.
Ndong et al. (2024) [29]	Senegal	Prospective cohort study	In‐theater laparoscopic appendectomy training evaluated via CUSUM analysis; standard 3‐port technique; and innovative use of low‐cost local instruments (e.g., glove endobag). Setting: Clinical and patient‐based training	81	Learning curve plateaued after 28 procedures; safe and effective training progression observed.
Nyundo et al. (2023) [30]	Multicountry (COSECSA region)	Cross‐sectional survey	Survey of training hospitals; explored equipment availability, simulation labs, curriculum integration, and faculty. Setting: Simulation and real patient‐based training	94 surgeons from 44 hospitals	Identified lack of trainers, equipment, and simulation labs as significant barriers; training is insufficient in most institutions.
Okrainec et al. 2009 [31]	Botswana	Controlled trial	Telesimulation‐based FLS training via Skype; weekly remote mentorship with interactive simulator sessions. Setting: Telesimulation and remote training	16	The telesimulation group had higher FLS scores and 100% pass rate vs. 38% in the self‐practice group; this demonstrated the feasibility of telesimulation.
Oosting et al. (2019) [32]	Mozambique	Descriptive report	Structured training model in Maputo Central hospital using animal models and mentorship from Dutch surgeons. Weekly laparoscopic procedures supported by team visits. Emphasis on stepwise training and practical exposure with local adaptation of techniques. Setting: Simulation and real patient‐based training	14 surgical trainees	Gradual increase in independent laparoscopic procedures; established baseline for further curriculum development in LMIC settings.
Reynolds et al. (2024) [33]	Malawi	Qualitative evaluation	Remote video‐based telementoring model. Participants performed basic laparoscopic procedures with live expert feedback using a smartphone and a low‐cost setup. Innovative in‐field mentorship using global linkups and real‐time procedural assistance. Setting: Telesimulation, remote training, and patient‐based training	10 general surgery residents	Improved confidence, increased exposure to laparoscopic cases; and demonstrated the feasibility of smartphone‐assisted remote surgical education.
Shreckengost et al. (2022) [34]	Bolivia	Mixed‐methods study	Virtual laparoscopic course during COVID‐19 using Zoom and WhatsApp. Combined didactics with low‐cost box trainers (< 15 USD), guided video submission, and small‐group mentoring. Prerecorded skill videos and live sessions are used in tandem. Setting: Telesimulation, remote training, and patient‐based training	24 (13 surgeons, 10 residents, 1 unspecified)	Significant improvement in test scores and skills (e.g., intracorporeal knot tying and precision cutting); 100% found course useful and would recommend.

Abbreviations: COSECSA, College of Surgeons of East, Central and Southern Africa; FLS, Fundamentals of Laparoscopic Surgery; LMIC, low‐ and middle‐income countries; OSAT, Objective Structured Assessment of Technical Skills; USD, United States Dollar.

### 3.2 Training Methods

**FIGURE 3:**
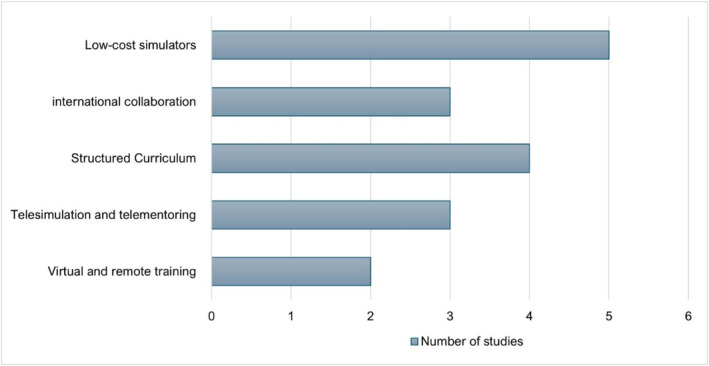
Methods used for training in the included studies.

A diverse range of training modalities was employed, often tailored to the realities of resource‐limited environments (Figure 3). Low‐cost simulation tools were widely used, including box trainers constructed from locally sourced materials [21, 29, 37], smartphone‐based simulators [29], and low‐cost suturing models developed during the COVID‐19 pandemic [30].

Telesimulation and telementoring approaches have proven effective as demonstrated by Skype‐based FLS training in Botswana [35], the Global Laparoscopic Advancement Program (GLAP) in Costa Rica and Mexico [23], and smartphone‐enabled real‐time mentoring in Malawi [36]. Structured curricula were reported in several settings, such as the longitudinal skills program in Ethiopia [31], a 3‐day gasless laparoscopy course in India [22], and integrated training within institutional simulation centers [24, 26]. Capacity‐building initiatives also relied on international partnerships, with successful examples in Ghana [27, 28], Brazil [32], and Mozambique [13].

### 3.3 Participants

The number and type of participants varied substantially across studies. Small‐scale pilot and feasibility studies typically enrolled between 7 and 20 participants [21, 22, 25, 29]. Larger training initiatives included 88 surgical residents in Ethiopia [31], 94 surgeons from 44 hospitals across the College of Surgeons of East, Central, and Southern Africa (COSECSA) region [34], and more than 12,000 participants trained over a decade at a Brazilian institution [24]. Participants were mainly surgical residents, but many programs also engaged consultants [33], house officers [21], nurses [28], and multidisciplinary surgical teams [13].

### 3.4 Outcomes

**FIGURE 4:**
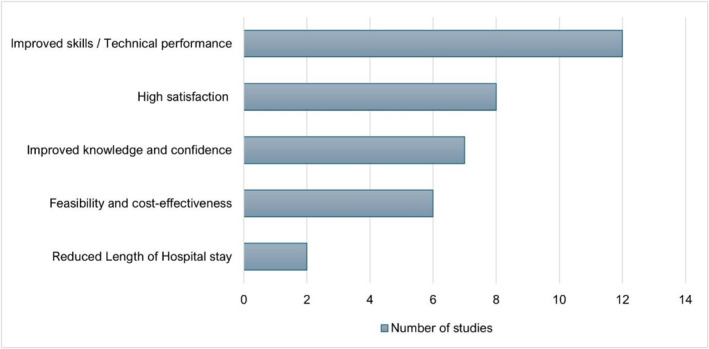
Methods used for assessment of outcomes of the laparoscopy training in the included studies.

All studies reported positive outcomes, with improvements in knowledge, confidence, or technical performance (Figure 4). Simulation‐based training resulted in measurable skill acquisition, including faster task completion and improved scores on validated assessment tools, such as the FLS and OSATS [17, 18, 25, 27]. Telesimulation programs have demonstrated strong efficacy, with significantly higher FLS pass rates compared to self‐directed practice [35] and enhanced surgical exposure, resulting in high satisfaction in Latin America and Malawi [23, 36].

Longitudinal implementation studies in Ghana have highlighted the progressive adoption of laparoscopic surgery, with an increasing number of local surgeons achieving independence, shorter hospital stays, and complication rates comparable to or lower than those of open procedures [27, 28]. Low‐cost and improvised training models were validated as effective alternatives to commercial simulators [25, 37].

Surveys and reviews revealed challenges to LMIC laparoscopic education, including a shortage of training equipment, limited access to simulation labs, and a lack of structured curricula [20, 32, 34]. Overall, the evidence demonstrates that laparoscopic training in LMICs is both feasible and effective, with high levels of participant satisfaction and clear potential for sustainable integration into surgical education systems.

### 3.5 Clinical Training and Patient Outcomes

A subset of studies reported training with direct patient care and clinical outcomes. In Ghana, longitudinal implementation programs demonstrated successful transition to independent laparoscopic practice, with increasing proportions of procedures performed by local surgeons and outcomes comparable to open surgery, including low complication rates and reduced hospital stay [27, 28]. Similarly, in Senegal, laparoscopic appendectomy training demonstrated a clear learning curve, with proficiency achieved after approximately 28 cases using cumulative sum analysis [33]. In Mozambique and Malawi, mentorship‐based and telementoring approaches facilitated gradual increases in independent procedures and improved clinical exposure [13, 36]. These findings suggest that training programs linked to real clinical practice are critical for sustainable adoption.

## 4 Discussion

With continual innovation and introduction of new surgical technologies and refinement of techniques, minimally invasive surgery has become the standard of care in many developed/high‐income countries [4]. Laparoscopy has been adopted across multiple surgical specialties and is used routinely to perform appendectomies, cholecystectomies, colectomies, and various urological and gynecological procedures. The benefits of this approach are well established and include reduced postoperative pain and analgesic requirements, earlier mobilization, a shorter hospital stay, and an earlier resumption of normal daily activity [38].

However, the benefits derived from minimally invasive surgery, which have the potential to transform and revolutionize healthcare in low‐ and middle‐income countries (LMICs), are not widely recognized due to resource limitations [14, 39]. Moreover, a lack of training opportunities, limited access to mentors and dedicated laparoscopic fellowships, stakeholder dynamics, time constraints, departmental structure, and a lack of institutional support are additional hurdles [40, 41].

These barriers require strategic and innovative solutions, along with a multifaceted approach, to improve laparoscopic training and provision in LMICs [39]. These begin with national investment in training programs, improved equipment availability and maintenance, and increased access to local trainers [41]. This, coupled with the development of cost‐effective training methods and a supportive environment for local trainees, will significantly contribute to achieving the defined goals.

This review of the literature included nineteen studies [13, 20, 21, 22, 23, 24, 25, 26, 27, 28, 29, 30, 31, 32, 33, 34, 35, 36, 37] that all describe innovative and successful methods used globally in LMICs to enhance familiarity and skills in laparoscopic training. These included countries mainly from Africa and Latin America, with a mixture of cohort sizes and study designs. Training methods included simulation‐based programs using locally sourced box trainers [21, 29, 37], virtual telesimulation [13, 35, 36], smartphone telementoring [24], allowing residents to perform tasks under real‐time supervision and support, in‐person training, and structured training using animal models and mentorship from visiting surgeons. Although most of the included studies demonstrate feasibility, several programs provide valuable insights into successful large‐scale implementation. The IRCAD program in Brazil trained over 12,000 participants over a decade, demonstrating the scalability of structured curricula integrated with simulation and clinical exposure [24]. Similarly, longitudinal programs in Ghana showed progressive adoption of laparoscopic surgery, with increasing local surgeon independence and excellent clinical outcomes [27, 28]. Key factors contributing to success included strong local leadership (‘surgeon champions’), sustained mentorship, integration of simulation with real operative experience, institutional support, and stepwise capacity building. These elements appear critical for transitioning from short‐term training interventions to sustainable national programs.

Funding is often cited as a significant obstacle to laparoscopic training in LMICs, and such procedures may be confined to a small number of large urban centers. To improve sustainability and cost‐effectiveness, modified procedures (such as gasless laparoscopy) may provide longer‐term solutions [22].

Moreover, telesimulation allows overcoming geographical barriers, potentially reducing costs associated with in‐person training [13, 23, 35, 36]. Remote supervision and assessment via video communication enable educators to facilitate learning, provide feedback, and make training programs accessible to broader audiences, particularly in remote and/or resource‐deprived areas.

Okrainec et al. [35] used telesimulation in a multicenter study involving 16 surgeons in Botswana to teach the fundamentals of laparoscopic surgery (FLS). The cohort was divided into two groups: a self‐practice group using a standard FLS simulator and a telesimulation group working under the guidance and instruction of an FLS proctor. Significantly, higher posttest FLS scores were observed in the telesimulation group compared with the self‐directed learning group. This method could be cost‐effective for reaching audiences in remote resource‐restricted locations. Similarly, this modality is an effective method for teaching other procedural and technical skills [42].

Paradigm shifts and novel ways of working during the COVID‐19 pandemic further emphasized the need to develop alternative methods of medical education and tutoring [30]. A feasibility study on teaching advanced laparoscopic suturing skills to residents through telesimulation was found to be effective [43]. Trainees found telesimulation to be easy to use, with a strong educational value that enhanced their skills and technical competence.

Similarly, Shreckengost et al. [37] developed a virtual basic laparoscopic surgery course taught through weekly live videoconferences (via Zoom and WhatsApp) and additional mentorship. They reported improved confidence, knowledge, and basic skills amongst participants from Bolivia. These platforms are not only cost‐effective but also offer continuity in training, especially during major disruptions.

Collaboration with experts from institutions where laparoscopy is routinely performed can result in effective laparoscopic skills training [31]. For instance, the GLAP initiative, developed by SAGES, was originally established as a week‐long in‐person simulation training program for surgeons in LMICs. This includes dry and wet labs, proctoring, and didactic sessions. In response to the COVID‐19 pandemic, GLAP was modified and delivered virtually and it was found to be an effective teaching strategy [23].

Training, costs, and equipment/infrastructure are generally the main challenges to the introduction of laparoscopic surgery in LMICs [40]. The general shortage of trained laparoscopic surgeons results in limited local training opportunities and educational resources. Consequently, methods, such as telesimulation, are important in facilitating the transfer of knowledge and skills between institutes [44].

Additionally, locally sourced and/or low‐cost laparoscopic simulators may also be useful teaching adjuncts. These have been developed from cardboard boxes and other recyclable materials [20, 45]. Validated training tools have been used to assess low‐cost laparoscopic box trainers, and these tools have been effective [45]. Recordings of commonly performed laparoscopic procedures are freely available online. These are potentially underutilized learning tools that can be better used to aid understanding and consolidate knowledge [46].

Other methods include the development of a low‐cost surgical trainer using iPhone technology [25]. Construction of the trainer took less than 1 hour, and the device allows surgeons in any location to practice their laparoscopic skills. Creative course structuring, such as the development of Grey's laparoscopic suturing course (GLSC), is feasible and can potentially benefit a large number of trainees in countries with limited resources and industry support [30].

Overall, a diverse range of global studies demonstrate that laparoscopic training in LMICs is both feasible and effective, with high levels of participant satisfaction. Creative solutions can help overcome challenges and obstacles, and these methods/programs have a clear potential to be integrated into worldwide surgical education systems and curricula.

### 4.1 Limitations

This review has several limitations. First, the heterogeneity of study designs, training methods, and reported outcomes precluded formal meta‐analysis, limiting quantitative synthesis. Second, most included studies were small pilot or feasibility studies, often lacking long‐term follow‐up or standardized outcome measures. Third, there is potential publication bias as successful interventions are more likely to be reported, whereas unsuccessful or discontinued programs may remain unpublished. Fourth, many studies relied on subjective outcomes, such as participant satisfaction, with relatively few reporting validated objective performance measures. Finally, regional representation was uneven, with a predominance of studies from sub‐Saharan Africa and Latin America, limiting generalizability to other LMIC regions such as Southeast Asia and the Middle East.

## 5 Conclusion

This systematic review demonstrates that laparoscopic training initiatives in LMICs are both feasible and effective, with consistent improvements in technical performance, knowledge, and trainee confidence. A range of innovative strategies, including low‐cost simulation, telesimulation, and structured curricula, have been successfully adapted to resource‐constrained environments. Despite these advances, significant barriers remain, particularly regarding access to equipment, simulation facilities, and integration into formal surgical curricula. Sustained investment, international collaboration, and incorporation of laparoscopic training into residency programs are essential to scale up capacity. Future research should prioritize standardized outcome measures, long‐term follow‐up, and evaluation of cost‐effectiveness to guide policy and program development.

## Author Contributions

**Ali Yasen Mohamedahmed:** conceptualization, investigation, writing–original draft, methodology, visualization, writing–review and editing, formal analysis, software, data curation, validation. **Shafquat Zaman:** writing–original draft, writing–review and editing. **Safeya Mohammed:** methodology, formal analysis. **Seddig Adam Jebril Saleh:** investigation, methodology. **Mohammed A. Adam:** methodology, writing–original draft, software. **Johnelize Louw:** conceptualization, writing–review and editing, project administration. **Tiffany E. Chao:** conceptualization, writing–review and editing, project administration, supervision, validation. **Kathryn Chu:** supervision, resources, writing–review and editing, conceptualization, validation.

## Funding

The authors have nothing to report.

## Ethics Statement

This study received exemption from ethical approval by the Health Research Ethics Committee, Stellenbosch University, South Africa.

## Conflicts of Interest

The authors declare no conflicts of interest.

## Data Availability

The data that supports the findings of this study are available in the supplementary material of this article.
